# Effect of Interpersonal Injustice on Knowledge Hiding Behavior: Moderating Role of High-Performance Work Stress

**DOI:** 10.3389/fpsyg.2022.858669

**Published:** 2022-04-04

**Authors:** Yi Cao

**Affiliations:** School of Management, Hebei Finance University, Baoding, China

**Keywords:** interpersonal justice, emotional exhaustion, evasive hiding, playing dumb, rationalized hiding, high-performance work stress

## Abstract

The underlying aim of this study was to investigate the impact of interpersonal injustice on emotional exhaustion and the three main facets of knowledge hiding, i.e., evasive knowledge hiding, playing dumb, and rationalized knowledge hiding. This study also investigates the moderating role of high-performance work stress in the relationship between interpersonal injustice and emotional exhaustion. A questionnaire was adopted to obtain data from 539 employees working in the telecom sector of China. The Smart-PLS software was used to analyze the data through the aid of a structural equation modeling (SEM) technique. The results revealed that interpersonal injustice had a positive and significant relationship with evasive knowledge hiding, playing dumb, and rationalized knowledge hiding. Interpersonal injustice also had a positive relationship with emotional exhaustion, and it was found that emotional exhaustion had a positive relationship with evasive knowledge hiding, playing dumb, and rationalized knowledge hiding. The results also revealed that emotional exhaustion mediated the relationship between interpersonal injustice and knowledge hiding (i.e., evasive hiding, playing dumb, and rationalized hiding). Moreover, it was also observed that high-performance work stress significantly but negatively moderated the relationship between interpersonal injustice and emotional exhaustion. Theoretically, this study made a valuable contribution by examining the impact of interpersonal injustice on knowledge hiding behavior. In terms of practical implications, this study would certainly aid the organizations to support a fair and just workplace culture that encourages knowledge sharing.

## Introduction

Organizational success depends on knowledge exchange. Workers’ desire to exchange information in the form of notions, expertise, reality, methods, or formulae with other people within the organization is referred to as knowledge sharing. People, groups, and organizations benefit from sustained knowledge sharing, which includes innovative work behavior, creative performance, team creativity, and knowledge management (Wang et al., 2011; Connelly et al., 2012; Dong et al., 2016). Workers are required to share this knowledge and information willingly with one another to ensure seamless organizational effectiveness. Knowledge sharing may be advantageous for individuals operating outside of organizational bounds, in addition to its applicability within organizational constraints (Mesmer-Magnus et al., 2011; Men et al., 2019). Given the success of information sharing, businesses frequently encounter the problem of knowledge hiding practices, which occur when an individual intentionally hides or conceals knowledge when it is required by someone else in the workplace.

Knowledge concealing practices may be noticed in the workplace in three different ways as follows: acting dumb, reasoned hiding, and evasive hiding (Huo et al., 2016; Bari et al., 2019b; Gerpott et al., 2019). Individuals may engage in such activities for a variety of reasons, including a fear of losing their position, influence, or wealth. Consequently, the frequency of knowledge-hiding behaviors at work is growing faster than the frequency of knowledge-sharing behaviors, with high levels of negative results (Holten et al., 2016; Connelly et al., 2019). Therefore, it is critical to learn more about knowledge-hiding practices and their implications. Every institution’s intellectual capital plays a critical function that might affect the productivity of businesses and workers. Unfortunately, owing to the habit of knowledge hiding, achieving satisfying outcomes in intellectual capital might be difficult. Workers are unwilling to share information for a variety of reasons, including knowledge ownership preservation and management, specialized supremacy, and protective consciousness (Connelly and Kevin Kelloway, 2003; Peng, 2013).

Nearly half of the workers want to suppress, misrepresent, or conceal information that has been sought by someone else. Knowledge concealing, which is distinct from the flip side of knowledge sharing, is the purposeful failure to provide essential knowledge to colleagues when requested (Zhao et al., 2019). Clearly, knowledge concealment decreases the effectiveness of the exchange of knowledge among members, impedes the production of new suggestions, and even destroys trust, raising the danger of knowledge loss and hindering personal and team innovation (Černe et al., 2014; Bogilović et al., 2017). In this line, solving the problem of insufficient information sharing by eliminating knowledge hiding and increasing knowledge conversion inside businesses makes sense (Xiaolong et al., 2021).

Many academics have begun to study the harmful repercussions of these actions since the conceptualization of knowledge concealment in the organizational setting happens. Creativity, originality, work behavior, and performance have all been demonstrated to be adversely correlated with knowledge concealing (Connelly and Zweig, 2015; Burmeister et al., 2018; Bari et al., 2019a; Wang et al., 2019). It has been discovered that withholding information weakens interpersonal connections and increases interpersonal distrust. According to many academics, employees who suppress their expertise on purpose are regarded as antisocial. The presence of such people in an organization has a negative influence on the working environment (Connelly and Zweig, 2015; Alnaimi and Rjoub, 2021). Several studies have shown that information concealment has a detrimental impact on innovation. Unfortunately, none of the prior investigations looked at the link between organizational unfairness and knowledge-hiding elements such as evasive concealment, playing dumb, and justified hiding.

Each aspect of information concealment has a unique scenario, and these scenarios might have varying effects (both good and bad) on knowledge researchers (Men et al., 2019). Evasive information hiding and acting dumb, for example, are both deceptive; however, a justified knowledge hider explains his/her role and justifies his/her knowledge concealing. Evasive concealment is a restricted trickery knowledge concealing activity in which the hider gives erroneous knowledge or a deceptive commitment to provide a comprehensive response in the future (which he or she has no intention of delivering). Playing dumb is a knowledge concealment trickery in which the lasher pretends to be unaware of the relevant knowledge or refuses to supply it. Rationalized hiding is a type of conditioned trickery knowledge concealment in which the suppressor justifies his or her refusal to divulge requested information by explaining why he or she is unable to do so, or by criticizing the second party (Connelly et al., 2012).

Workers who keep their knowledge hidden are a big danger to both individual and organizational success in competitive environments. The deliberate effort to withhold or conceal knowledge that has been sought by another person is known as knowledge concealment. Evasive hiding, acting stupid, and justified hiding are the three interconnected techniques. Employee innovation can be stifled, and corporate growth and competitiveness can be harmed as a result of such activities (Bogilović et al., 2017; Rhee and Choi, 2017; Singh, 2019; Wang et al., 2019). According to a previous study, it is caused by showing orientation, inherent competition, possessiveness, personality management, leader-signaled information concealing, rising unemployment, time demands, interpersonal mistrust, professional ostracism, organizational factors, or psychological empowerment (Serenko and Bontis, 2016b; Zhao et al., 2016; Škerlavaj et al., 2018; Hernaus et al., 2019; Malik et al., 2019; Offergelt et al., 2019; Peng et al., 2019).

Another important factor that may encourage knowledge concealment is organizational unfairness or workers’ perceptions of unjust treatment by their employers. Staff members may believe that they are being treated unfairly if they believe their rewards are not commensurate with their contributions, as measured by work assignments, pay, bonuses, evaluations, and promotions, if they are not allowed to express their opinions, or if they believe organizational authorities do not treat them with dignity and respect. Interpersonal injustice, as a negative working experience is widespread and significant in a variety of cultures. Even though some previous research work suggests a link among employees’ experiences of organizational injustice, such as interpersonal unfairness and knowledge concealment (Barclay and Saldanha, 2016; Lavelle et al., 2018; Lu and McKeown, 2018; Khattak et al., 2019).

The probable antecedent has gotten a lot of research but has not gotten a lot of attention. It is also unclear why and how sentiments of unfairness could lead to increased knowledge-hiding actions (Zagenczyk et al., 2013). To fill this gap, this research looks at the understudied possibly causal process of organizational unfairness. Employees may mentally separate themselves from an organization as they perceive to be unfair, which can lead to undesirable job outcomes such as unethical behavior and the expression of strong unfavorable sentiments, or plans to resign. Adding to previous research, this study proposes that exposure to interpersonal injustice may cause knowledge hiding due to organizational misidentification, but that process may be exacerbated by high-performance work stress, which means that employees’ outcomes are worsened even when they put in more effort (Nguyen and Leclerc, 2011; Ning and Zhaoyi, 2017).

To identify the impact of interpersonal injustice as a dimension of organizational injustice suggested by Jahanzeb et al. (2021), this study has some questions to address, such as how interpersonal injustice would lead to knowledge hiding? What factors will worsen or mediate the relationship between interpersonal injustice and knowledge hiding? What moderation would affect these relationships? This research was designed to address the unaddressed research questions in the past with certain objectives, such as analyzing the impact of interpersonal injustice on knowledge hiding behaviors, evaluating the mediating role of emotional exhaustion, and exploring the moderating role of high-performance work stress during the process of knowledge hiding behaviors.

## Theoretical Framework and Hypothesis Development

This research revolves around identifying the effects of interpersonal injustice on factors of knowledge hiding, such as playing dumb, rationalized hiding, and evasive hiding. The mediating role of emotional exhaustion was also studied in this research along with moderated mediation of high-performance work stress. This research model was based on certain theories given below.

In an organizational environment, social exchange theory offers a useful theoretical relationship between organizational justice and individual reactions. The main premise of social exchange theory would be that human relationships evolve into reciprocal commitments over time and that obligations are impacted by numerous exchange norms. The reciprocity principle is the most influential principle. The behavior of a person participating in trade activities within a social system is explained by social exchange theory, which is a common basis for comparison. This implies that all participants in the social system have something that the other participants appreciate and that they likewise demand something worthwhile from other members. Relationships between sociological members are two-way reciprocal transactions that are based on the anticipation of prospective benefits from each other. Individuals, according to social exchange theory, are motivated by their own self-interest. Each act of providing something of value should elicit a response from the receiver, laying the groundwork for a mutually beneficial exchange process (Homans, 1958, 1961; Back, 1965).

The notion of social exchange has been used to study a variety of human behaviors, particularly information sharing. Workers share information with other colleagues since they anticipate getting anything of value back, including current and future returns. To put it another way, one employee may share his or her expertise with another only after negotiating or assuming that the other employee will similarly share his or her information with him/her when required. There are both theoretically and empirically available arguments, suggesting that reciprocation plays a crucial role in information sharing behavior. However, people may respond not only to good but also to harmful behaviors (Liu et al., 2011; Lin and Lo, 2015; Serenko and Bontis, 2016a).

This study uses social identity theory to explain the theoretical reasons concerning the indirect influence of perceived organizational unfairness on knowledge concealment, through emotional weariness, and the increasing role of high-performance job stress. As per this hypothesis, individuals enter many social groups (e.g., ethnicity, religious background, age group, and organizations) in order to integrate into their social environment and alleviate social ambiguity (Adams, 1965; Tajfel and Turner, 2004; Lovegrove and Fairley, 2016; Ambrose et al., 2018). They gain some self-confidence through generating positive assessments of the groupings toward which individuals adhere and negative judgments of categories with which they have no relationship (e.g., rival sports teams) (Jahanzeb et al., 2021). Employees may also mentally distance themselves from their employer if they do not agree (Todd and Kent, 2009; Lai et al., 2013; Zagenczyk et al., 2013).

According to referent cognition theory, whenever a person is subjected to relative deprivation, he or she will experience wrath and hatred. The severity of rage is determined by the following three factors: the reference outcome, the anticipation of such a future outcome, and the reason. A high reference outcome, which is a poor prediction of future outcomes, and a low justification for the incident all contribute to the emotion of resentment. Several empirical studies in a laboratory context have demonstrated the impact of these three referent cognition theory components (Folger, 1984; Aquino et al., 1997; Colquitt and Shaw, 2005). This theory provided the basis for understanding the outcomes of interpersonal injustice leading to knowledge hiding in the organizational context.

### Interpersonal Injustice, Knowledge Hiding, and Emotional Exhaustion

Organizational justice encompasses the equal treatment, distributive and procedural, and interactional justice, as well as the sense of fairness with which top management conducts organizational operations. Interactional justice refers to the perceived fairness in day-to-day encounters with supervisors and is more significant to immediate superiors and other authority figures. Interactional justice encompasses different types of social justice as follows: interpersonal and informative. Interpersonal justice relates to bosses’ decent treatment of subordinates, where supervisors convey in their interactions with subordinates through civility, decency, and sincerity, which subordinates view as a confirmation of their organizational status. Staff should be trained about why certain processes were followed, which is known as informational justice (Greenberg, 1990; Colquitt, 2001; Bies, 2005; Hameed et al., 2019). Every individual is obliged to treat coworkers with respect and dignity at the very least. Such a type of behavior is anticipated not only while communicating about a proposal’s conclusion but also on a day-to-day routine. In a similar vein, this methodology utilizes the notion of interpersonal injustice, which refers to how colleagues are treated with no decency and respect.

Since it is commonly established that such an action elicits a response, if an employee treats a coworker with disrespect and dignity, the coworker will elicit a response, even if it is not instantaneous. When we meet up with other humans, we always bring up recollections of previous meetings. If that is the situation, the colleague will engage in increased information concealment activity. As a result, a lack of interpersonal fairness among colleagues, as well as one having expertise authority over another, may contribute to knowledge concealment. It has been noticed that it affects both the hider and the target’s inventiveness and creativity. The social exchange hypothesis might be used to describe the entire connection. It should be highlighted that in the situation of “tacit” information, this behavior would be more obvious (Jahanzeb et al., 2021). Several studies have found organizational injustice including interpersonal injustice that impacts knowledge hiding behaviors (Fatima et al., 2021). Similarly, impacts of interpersonal injustice have been reported on the emotional exhaustion of the workers (Howard and Cordes, 2010). Therefore, we proposed the following hypotheses:

H_1._ *Interpersonal injustice has an effect on evasive knowledge hiding behavior.*
H_2._ *Interpersonal injustice has an effect on playing dumb.*
H_3._ *Interpersonal injustice has an effect on rationalized knowledge hiding behavior.*
H_4._ *Interpersonal injustice has an effect on emotional exhaustion.*

### Emotional Exhaustion, Knowledge Hiding, and Its Mediating Role

Emotional exhaustion refers to physical and mental tiredness produced by an individual’s failure to meet job demands, which can result in psychophysiological symptoms including despair and anxiety. Individual emotions modulate the effect of job events on actions. Furthermore, researchers suggested that emotional exhaustion may have a positive impact on knowledge concealment. First, the effective event hypothesis says that particular emotions might cause people to behave in a specific way. Employees who are emotionally weary may feel physically and psychologically exhausted, without direction and enthusiasm, and be uninterested in organizational matters (Maslach, 1993; Weiss and Cropanzano, 1996; Blanco–Donoso et al., 2017). In turn, an empirical study shows that such workers are more likely to express negative feelings *via* violent and deviant actions, such as refusing to cooperate with coworkers on purpose. Employees who are emotionally exhausted are more likely to hide their expertise. Second, researchers have found that unpleasant emotions have a direct link to unproductive job conduct (Eissa and Lester, 2017; Wolf et al., 2017; Thompson et al., 2019).

Finally, unfavorable work attitudes resulting from emotional weariness might send out dangerous signals. When others ask for expertise, employees may have a bad impression of their competitive environment. They may prefer information concealment because they are afraid of losing control over their knowledge and work, jeopardizing their competitive advantages, and even losing bargaining power with the business. It is easy to see how emotional exhaustion mediates the effect of interpersonal injustice on knowledge concealment if they follow the affective event theory order of “event to emotional reactions to conduct.” Poorly balanced interpersonal injustice (work environment characteristic) can lead to stress (work event), which causes employees to become emotionally exhausted (emotional reaction), prompting them to conceal information when confronted with each other’s demands (behavior) (Zhao and Jiang, 2021). Therefore, drawn from this literature support, we proposed the following hypotheses:

H_5._ *Emotional exhaustion has an effect on evasive knowledge hiding behavior.*
H_6._ *Emotional exhaustion has an effect on playing dumb.*
H_7._ *Emotional exhaustion has an effect on rationalized knowledge hiding behavior.*
H_8._ *Emotional exhaustion mediates the relationship of interpersonal injustice and evasive hiding.*
H_9._ *Emotional exhaustion mediates the relationship of interpersonal injustice and playing dumb.*
H_10._ *Emotional exhaustion mediates the relationship of interpersonal injustice and rationalized hiding.*

### Moderating Effects of High–Performance Work Stress

Workplace stress relates to a person’s subjective experience of a gap between how much they can acquire and how much they should spend to satisfy expectations, as well as the potential for negative effects of such a gap. High job expectations typically accompany the resources given by high–performance work systems in businesses, which can contribute to greater employee stress. The high–performance work system, in contrast to standard human resource management approaches, stresses employee engagement and autonomy. Employees feel increased job expectations as a result of this authorization, which also comes with independent decision–making duties (Selye, 1976; Ramsay et al., 2000; Jensen et al., 2011). Second, a high–performance work structure ties pay and advancement to results. To guarantee their competitiveness in the firm, workers must enhance their job efficiency and devote more time and energy to it, which raises the perceived assessment methods.

Even though a high–performance work system raises employee performance management requirements, it will neither place a premium on quantitative work nor establish clearly defined objectives. Workers must expend quite a lot of energy in order to receive a better assessment, therefore, raising the perceived job demands. When they are faced with high job demand, workers say that the effort expended (including working long hours and effort) is insufficient to satisfy high job demands (Escribá Carda et al., 2016; Han et al., 2020). According to an empirical study, employee wellbeing is also reduced by job stress. For instance, when people are under a lot of stress at work, their pleasant emotional experience decreases. According to several studies, when employees are under a lot of stress at work, their psychological resources are quickly depleted, resulting in negative sensations such as anxiety and emotional tiredness (Siu, 2002; Liu et al., 2020). Moderating effects of high–performance work stress were also studied by Golparvar et al. (2012) and got significant results of moderation toward emotional exhaustion. Therefore, the following hypothesis was suggested:

H_11._ *High–performance work stress moderates the relationship between interpersonal injustice and emotional exhaustion.*

**FIGURE 1:**
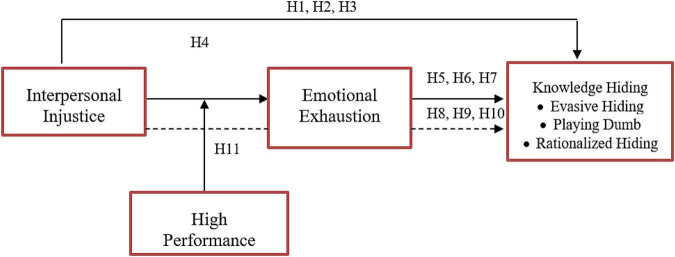
Theoretical framework. INJ, interpersonal justice; EE, emotional exhaustion; EH, evasive hiding; PD, playing dumb; RH, rationalized hiding; WS, high-performance work stress.

A following conceptual framework (Figure 1) has been formed based on the above literature and hypothesis.

## Methodology

The validation of the hypothesis for this study was carried out using a quantitative research design along with the deductive approach. The hypothesis for the study helped to examine the effect of precursors or predictors on outcome variables. Elimination of any biases from the study was made possible due to the use of this research design. The data for this quantitative study were collected through the use of a self–administered survey. The rationality of the data was ensured by making the items of each variable clear and short. Also, the respondents were asked to stay relaxed and be natural while filling out the questionnaires as there were no correct or incorrect answers. The target population was the employees who were working in the telecommunication sector. This study deploys a convenience sampling technique in order to sample from the target population. This technique helped the researcher to acquire data from the study participants cost–effectively and efficiently (Nawaz et al., 2020; Avotra et al., 2021; Yingfei et al., 2021). The convenience sampling technique also helped to collect data from the participants who were readily and conveniently available. The sample size for this study was 539. The unit of analysis was the employees of the telecommunication sector of China.

### Statistical Tool

The data were analyzed using Smart–PLS 3.3.3 software. This software helped to analyze the structure equation technique (SEM). Also, the deep insights of small data were gained through this software (Bari et al., 2019b). Moreover, Smart–PLS allowed the researcher to develop a path model in a few minutes; therefore, it is an efficient tool. There are two stages through which the data are analyzed using this software. The measurement model, which is the first stage, includes data validity (i.e., factor loadings, average variance extracted (AVE), Heterotrait–Monotrait (HTMT), and Fornell and Larker criterion) and data reliability (i.e., Cronbach’s alpha and composite reliabilities) (Nawaz et al., 2019). The structural model is the second stage through which the hypothesis is either supported or not supported. These are analyzed using *p*–values, SD, sample means, and *F*–values.

### Measurement

A 5–point Likert scale was used to obtain data for each item of the variable under study. The reliability of each variable using the Cronbach’s alpha should be more than 0.70 (Sarstedt et al., 2019).

#### Interpersonal Injustice

The items for interpersonal justice were adopted from Skarlicki et al. (2008) comprised 4 items. The Cronbach’s alpha for this variable is 0.900; therefore, this variable is reliable.

#### Emotional Exhaustion

The items for emotional exhaustion were adopted from the Maslach Burnout Inventory by Piko (2006) that comprised 8 items. The Cronbach’s alpha for this variable is 0.935; therefore, this variable is reliable.

#### Knowledge Hiding

The three facets of knowledge hiding (i.e., evasive hiding, playing dumb, and rationalized hiding) were taken to measure knowledge hiding. This variable comprised a total of 12 items (4 items for evasive hiding, 4 items for playing dumb, and 4 items for rationalized hiding). All these items were adopted from Connelly et al. (2012). The Cronbach’s alpha for evasive hiding is 0.897; therefore, this variable is reliable. The Cronbach’s alpha for playing dumb is 0.929; therefore, this variable is reliable. The Cronbach’s alpha for rationalized hiding is 0.929; therefore, this variable is reliable.

#### High–Performance Work Stress

The items for high–performance work stress were adopted from Shukla and Srivastava (2016) that comprised 9 items. The Cronbach’s alpha for this variable is 0.918; therefore, this variable is reliable.

### Demographic Details

Table 1 shows the demographic profile of the employees who were the study participants. The total participants of this study were 539 and out of these, 64.19% were men and 35.18% were women. The employees between the age of 20 and 30 years were 41.93, between 31 and 40 years were 36.73, between 41 and 50 years were 15.58%, and above the age of 50 years were 5.75%. Out of the total number of participants, the bachelor degree holders were 50.28%, master’s degree holders were 31.35%, and the employees who had Ph.D. or other degrees were 18.37%. Furthermore, the employees with an organizational tenure of less than a year were 46.20%, the employees with an organizational tenure of between 1 and 3 years were 40.63%, the employees with an organizational tenure of between 4 and 6 years were 7.98%, while the employees with an organizational tenure of more than 6 years were 5.19%.

**TABLE 1:** Demographic analysis.

Demographics	Frequency	Percentage
**Gender**		
Male	346	64.19%
Female	193	35.81%
**Age (years)**		
20–30	226	41.93%
31–40	198	36.73%
41–50	84	15.58%
Above 50	31	5.75%
**Education**		
Bachelors	271	50.28%
Masters	169	31.35%
Ph.D. and others	99	18.37%
**Organizational tenure (years)**		
Less than 1	249	46.20%
1–3	219	40.63%
4–6	43	7.98%
More than 6	28	5.19%

*N = 539.*

### Common Method Bias

Table 2 shows the total variance explained of each item of the variables which is examined through single–factor analysis. It explains the common method bias, i.e., biasness of the questionnaire. The% of the variance for one item must be less than 50% (Yong and Pearce, 2013). The outcome for the total variance explained in this study is less than 50%; therefore, biasness is not present in the data.

**TABLE 2:** Total variance explained.

Component	Initial eigenvalues			Extraction sums of squared loadings		
	Total	% Of variance	Cumulative %	Total	% of Variance	Cumulative %
1	16.373	49.616	49.616	16.373	49.616	49.616
2	3.060	9.271	58.887			
3	2.403	7.282	66.169			
4	1.312	3.976	70.145			
5	1.159	3.514	73.659			
6	0.977	2.959	76.618			
7	0.827	2.506	79.124			
8	0.747	2.263	81.387			
9	0.697	2.112	83.499			
10	0.629	1.907	85.406			
11	0.586	1.776	87.182			
12	0.523	1.585	88.767			
13	0.466	1.412	90.178			
14	0.442	1.338	91.516			
15	0.398	1.206	92.722			
16	0.389	1.178	93.900			
17	0.345	1.046	94.946			
18	0.333	1.009	95.955			
19	0.275	0.835	96.789			
20	0.256	0.777	97.566			
21	0.192	0.582	98.149			
22	0.172	0.521	98.670			
23	0.110	0.333	99.003			
24	0.076	0.230	99.234			
25	0.056	0.170	99.404			
26	0.050	0.152	99.556			
27	0.038	0.115	99.672			
28	0.028	0.085	99.756			
29	0.024	0.073	99.829			
30	0.021	0.062	99.891			
31	0.017	0.053	99.944			
32	0.014	0.042	99.986			
33	0.005	0.014	100.000			

*Extraction method: principal component analysis.*

## Data Analysis and Results

### Measurement Model

**FIGURE 2:**
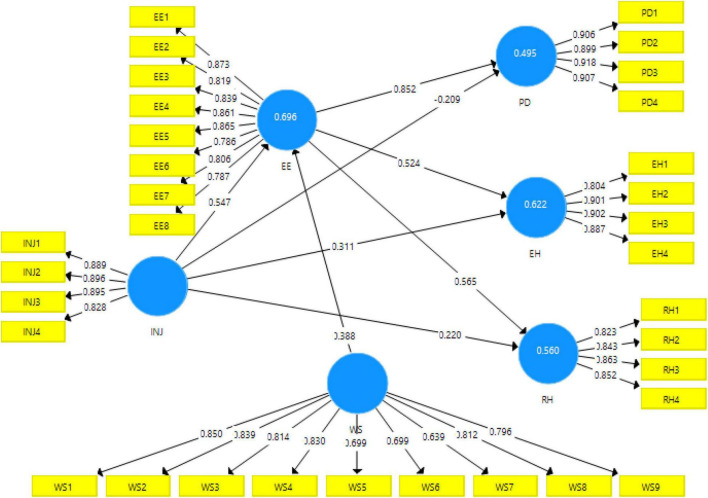
Output of measurement model algorithm.

The output measurement model algorithm can be seen in Figure 2. This figure explains the contribution of independent variables to the dependent variables of the study.

Table 3 presents factor loadings of each item of the study construct, i.e., interpersonal injustice, emotional exhaustion, knowledge hiding (i.e., evasive hiding, playing dumb, and rationalized hiding), and high–performance work stress. The table also shows the composite reliability and AVE along with the variance inflation factor (VIF) values. The factor loading explains the contribution of an item toward the variable, and its value must be greater than 0.60 (Jordan and Spiess, 2019). The factor loadings for all the items of this study are greater than 0.60; thus, the factor loadings are fair. VIF verifies the model’s collinearity issues. According to the study by Cho et al. (2020), the value of inner and outer VIFs should be less than 5, indicating that there is no issue of collinearity in the model. Moreover, the result of outer VIF for this study is also less than 5 (between 1.804 and 4.211), indicating that there is no issue of collinearity in the model. Moreover, the result of inner VIF for the present is also less than 5 (between 1.508 and 2.475). Table 3 also shows that the AVE values are more than 0.60; therefore, it indicates the existence of convergent validity (An et al., 2021). The composite reliability came out to be more than 0.70; therefore, it lies under the range of highly satisfactory value (Peterson and Kim, 2013).

**TABLE 3:** Model assessment (direct model).

Variables	Factor loadings		VIF	Composite reliability	AVE
Interpersonal injustice	INJ1	0.886	2.851		
	INJ2	0.895	2.893	**0.934**	**0.779**
	INJ3	0.904	3.191		
	INJ4	0.844	2.203		
Emotional exhaustion	EE1	0.887	4.512		
	EE2	0.821	2.945		
	EE3	0.848	3.670		
	EE4	0.861	3.846	**0.949**	**0.698**
	EE5	0.862	3.492		
	EE6	0.790	2.779		
	EE7	0.821	3.021		
	EE8	0.791	2.879		
Evasive hiding	EH1	0.819	1.902		
	EH2	0.899	3.012	**0.932**	**0.776**
	EH3	0.908	3.350		
	EH4	0.895	3.025		
Playing dumb	PD1	0.919	3.501		
	PD2	0.900	3.203	**0.953**	**0.834**
	PD3	0.919	3.929		
	PD4	0.916	3.663		
Rationalized hiding	RD1	0.835	1.914		
	RD2	0.857	2.192	**0.915**	**0.728**
	RD3	0.859	2.413		
	RD4	0.863	2.417		
					

*The bold value shows significance of the variables and relationship.*

Discriminant validity was examined using HTMT ratio and Fornell and Larker criteria as depicted in Table 4. These tests explain whether there is a difference between the variables or not. According to Xiaolong et al. (2021), the HTMT ratio should be less than 0.90 in order to ensure the discriminant validity of a variable. The result for the HTMT ratio for this study came out to be less than 0.90; therefore, discriminant validity is present.

**TABLE 4:** Discriminant validity (HTMT ratio).

	EE	EH	INJ	PD	RH	WS
EE						
EH	0.825					
INJ	0.828	0.791				
PD	0.746	0.432	0.488			
RH	0.810	0.711	0.743	0.534		
WS	0.736	0.625	0.629	0.470	0.767	

*INJ, interpersonal justice; EE, emotional exhaustion; EH, evasive hiding; PD, playing dumb; RH, rationalized hiding; WS, high–performance work stress.*

According to the study by Henseler et al. (2015), the criteria for Fornell and Larker criteria are met if the value at the top of the column is higher than the value below that column. Table 5 shows that the values at the top of the column are higher than the values below that column; therefore, discriminant validity is present.

**TABLE 5:** Discriminant validity (Fronell and Larcker criteria).

	EE	EH	INJ	PD	RH	WS
EE	0.830					
EH	0.764	0.874				
INJ	0.772	0.715	0.878			
PD	0.691	0.396	0.449	0.908		
RH	0.735	0.628	0.657	0.481	0.845	
WS	0.705	0.579	0.580	0.454	0.700	0.779

*INJ, interpersonal justice; EE, emotional exhaustion; EH, evasive hiding; PD, playing dumb; RH, rationalized hiding; WS, high–performance work stress.*

The *R*–squared value of more than or approximately 0.50 signifies that the model is substantially good (Archer et al., 2021). The *R*–squared values for the variables of this study are more than or approximately 0.50, which means that the model is good as shown in Table 6. The cross–validated redundancy measured from *Q*–square should be greater than zero (Henseler et al., 2015). The *Q*–squared values for the variables of this study are greater than zero; therefore, the model is significant.

**TABLE 6:** *R*–squared values and *Q*–squared values for the variables.

	R–Square	Q–Square
EE	0.696	0.438
EH	0.622	0.436
PD	0.495	0.373
RH	0.560	0.367

*INJ, interpersonal justice; EE, emotional exhaustion; EH, evasive hiding; PD, playing dumb; RH, rationalized hiding.*

### Structural Model

**FIGURE 3:**
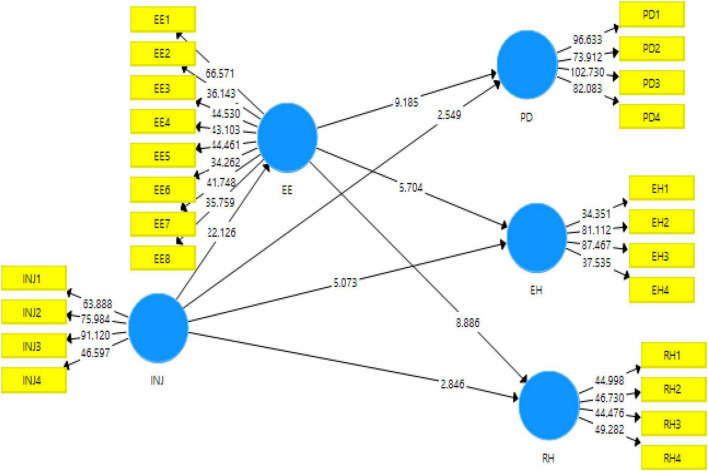
Structural model without moderation. INJ, interpersonal justice; EE, emotional exhaustion; EH, evasive hiding; PD, playing dumb; RH, rationalized hiding; WS, high–performance work stress.

The structural model was used to test the hypothesis of the study using *p*-value and *f*-value. The PLS-SEM bootstrapping model (refer to Figure 3) shows the validation of the proposed hypothesis. The relationships of the study were examined through the use of 95% corrected bootstrap.

**FIGURE 4:**
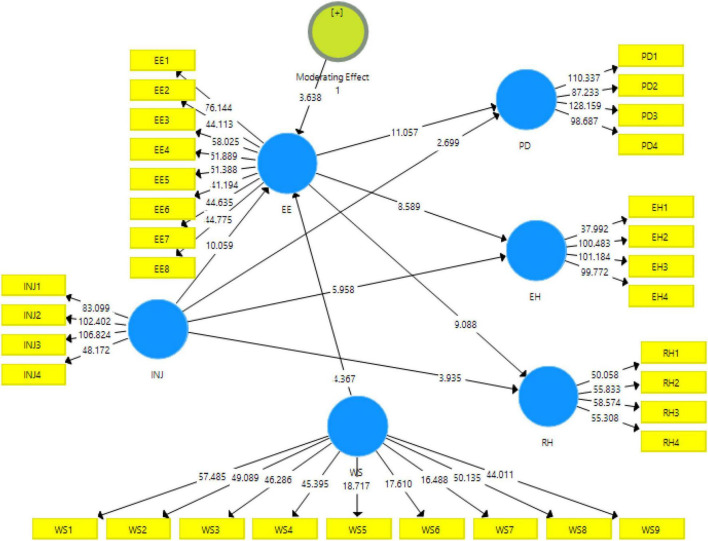
Structural model with moderation.

Table 7 shows the direct effect of the variables under study, Table 8 shows the indirect effects of the variable, and Table 9 and Figure 4 shows the moderating effects of the variables under study. These tables depict the acceptance or rejection of the hypothesis based on *p*-values that should be below 0.05 (Andrade, 2019). These tables also present the *f*^2^ value that explains the strength of the model, i.e., the value of approximately 0 has low strength and values of approximately 1 have greater strength (Bari et al., 2019b).

**TABLE 7:** Direct effects of the variable.

Paths	H	*O*	*M*	*SD*	*t*–statistics	*F* ^2^	*P*–value	Results
INJ → EH	H_1_	0.809	0.81	0.037	22.126	0.809	0.000***	***Accepted***
INJ → PD	H_2_	0.376	0.378	0.074	5.073	0.376	0.007***	***Accepted***
INJ → RH	H_3_	–0.249	–0.258	0.098	2.549	–0.249	0.000***	***Accepted***
INJ → EE	H_4_	0.192	0.199	0.068	2.846	0.192	0.000***	***Accepted***
EE → EH	H_5_	0.458	0.456	0.08	5.704	0.458	0.000***	***Accepted***
EE → PD	H_6_	0.929	0.941	0.101	9.185	0.929	0.000***	***Accepted***
EE → RH	H_7_	0.633	0.626	0.071	8.886	0.633	0.000***	***Accepted***

***p < 0.001.

*H, hypothesis; O, original sample; M = sample mean; SD, standard deviation; INJ, interpersonal justice; EE, emotional exhaustion; EH, evasive hiding; PD, playing dumb; RH, rationalized hiding. The bold value shows significance of the variables and relationship.*

**TABLE 8:** Indirect effects of the variable.

Paths	H	O	M	SD	*t*-statistics	*P*-value	Results
INJ → EE → EH	H_8_	0.371	0.368	0.062	6.025	0.000***	***Accepted***
INJ → EE → PD	H_9_	0.751	0.763	0.100	7.483	0.000***	***Accepted***
INJ → EE → RH	H_10_	0.512	0.506	0.053	9.592	0.000***	***Accepted***

*N = 539.*

***p < 0.001.

*O, original sample; M = sample mean; SD, standard deviation; INJ, interpersonal justice; EE, emotional exhaustion; EH, evasive hiding; PD, playing dumb; RH, rationalized hiding. The bold value shows significance of the variables and relationship.*

**TABLE 9:** Measurement model (moderation).

Variables	Factor loadings		VIF	Composite reliability	AVE
Interpersonal injustice	INJ1	0.889	2.851		
	INJ2	0.896	2.902	0.903	0.770
	INJ3	0.895	2.957		
	INJ4	0.828	2.032		
Emotional exhaustion	EE1	0.873	4.514		
	EE2	0.819	2.955		
	EE3	0.839	3.925		
	EE4	0.861	4.211	0.947	0.689
	EE5	0.865	3.762		
	EE6	0.786	2.833		
	EE7	0.806	2.940		
	EE8	0.787	2.795		
Evasive hiding	EH1	0.804	1.804		
	EH2	0.901	2.998	0.928	0.765
	EH3	0.902	3.241		
	EH4	0.887	2.927		
Playing dumb	PD1	0.906	3.065		
	PD2	0.899	3.161	0.949	0.924
	PD3	0.918	4.052		
	PD4	0.907	3.544		
Rationalized hiding	RD1	0.823	1.872		
	RD2	0.843	2.056	0.909	0.719
	RD3	0.863	2.503		
	RD4	0.852	2.372		
	WS1	0.850	3.606		
High Performance work stress	WS3	0.839	3.980		
	WS4	0.814	2.570	0.932	0.606
	WS5	0.830	2.581		
	WS6	0.699	1.904		
	WS7	0.699	2.405		
	WS8	0.639	2.015		
	WS9	0.812	3.577		

*INJ, interpersonal justice; EE, emotional exhaustion; EH, evasive hiding; PD, playing dumb; RH, rationalized hiding; WS, high-performance work stress.*

The first hypothesis (H_1_) was accepted as (*p* < 0.05); therefore, interpersonal injustice has a positive effect on evasive knowledge hiding behavior among the employees. *F*^2^ = 0.103 between these variables indicates that the effect size is small. The second hypothesis (H_2_) was accepted as (*p* < 0.05); therefore, interpersonal injustice has a positive effect on playing dumb among the employees. *F*^2^ = 0.035 between these variables indicates that the effect size is very small. The third hypothesis (H_3_) was accepted as (*p* < 0.05); therefore, interpersonal injustice has a positive effect on the rationalized knowledge hiding behavior among the employees. *F*^2^ = 0.045 between these variables indicates that the effect size is very small. The fourth hypothesis (H_4_) was accepted as (*p* < 0.05); therefore, interpersonal injustice has a positive effect on emotional exhaustion among the employees. *F*^2^ = 0.652 between these variables indicates that the effect size is large. The fifth hypothesis (H_5_) was accepted as (*p* < 0.05); therefore, emotional exhaustion has a positive effect on evasive knowledge hiding behavior among the employees. *F*^2^ = 0.294 between these variables indicates that the effect size is medium. The sixth hypothesis (H_6_) was accepted as (*p* < 0.05); therefore, emotional exhaustion has a positive effect on playing dumb among the employees. *F*^2^ = 0.581 between these variables indicates that the effect size is large. The seventh hypothesis (H_7_) was accepted as (*p* < 0.05); therefore, emotional exhaustion has a positive effect on rationalized knowledge hiding behavior among the employees. *F*^2^ = 0.062 between these variables indicates that the effect size is very small.

Table 8 shows the mediating effect of emotional exhaustion in the relationship between interpersonal injustice and knowledge hiding (i.e., evasive hiding, playing dumb, and rationalized hiding). The result shows that emotional exhaustion in the relationship between interpersonal injustice and knowledge hiding (evasive hiding) as *p* < 0.05. The result shows that emotional exhaustion in the relationship between interpersonal injustice and knowledge hiding (playing dumb) as *p* < 0.05. Also, the result shows that emotional exhaustion in the relationship between interpersonal injustice and knowledge hiding (rationalized hiding) as *p* < 0.05.

Before proceeding to the moderation of the high-performance work stress variable on the relationship of interpersonal injustice and emotional exhaustion, the data were further validated with the addition of the moderating variable considering reliabilities and validities of the scales. The results obtained had shown that the scales have been showing the values of reliability above the threshold of 0.7 (Hair et al., 2017). Similarly, the HTMT ratios and the values of the Fornell and Larcker criteria were also under the maximum threshold. The results for the moderation have been reported in Table 9.

The last hypothesis was accepted as *p* < 0.05; therefore, high-performance work stress moderates the relationship between interpersonal injustice and emotional exhaustion as shown in Table 10.

**TABLE 10:** Moderating effects of the variable.

Paths	H	*O*	*M*	*SD*	*t*-statistics	*P*-value	Results
Inj × WS → EE	H_11_	–0.139	–0.143	0.038	3.638	0.000***	***Accepted***

***p < 0.001.

*H, hypothesis; O, original sample; M = sample mean; SD, standard deviation; INJ, interpersonal justice; WS, high-performance work stress; EE, emotional exhaustion.*

*The bold value shows significance of the variables and relationship.*

## Discussion

This research focused on finding the impact of interpersonal injustice on knowledge hiding behaviors. This was previously studied by Jahanzeb et al. (2021). Organizational injustice has been clearly studied before impacting the behaviors of knowledge hiding in organizations. This study focused on the dimension of organizational injustice, which is interpersonal injustice toward hiding knowledge. Interpersonal injustice is the matter of injustice among individuals who could be colleagues, workers, or bosses and employees. Previously, a lot of research had been carried out on organizational justice and knowledge sharing, but very few had evaluated the impacts of injustice on knowledge hiding (Fatima et al., 2021). These researchers found significant relationships between the injustices at organizational and interpersonal levels and found significant associations. The first three hypotheses of this research also gave similar results, indicating a strong association between interpersonal injustice and factors of knowledge hiding.

The factors of knowledge hiding were playing dumb, evasive hiding, and rationalized hiding. The association of interpersonal injustice with all these three factors was strong, and all three hypotheses indicated that their relationships were accepted. These kinds of direct relationships were not formerly tested in prior research (Lanke, 2018; Fatima et al., 2021). The fourth hypothesis was about the relationship of interpersonal injustice with emotional exhaustion. Emotional exhaustion is a type of exhaustion that is related to work, and similar kinds of results were also obtained in some of the previous research (Howard and Cordes, 2010). These kinds of relationships could also lead to employee withdrawal as suggested by Howard and Cordes (2010).

The next 3 hypotheses dealt with the direct relationship of emotional exhaustion with evasive knowledge hiding, playing dumb, and rationalized knowledge hiding. All three hypotheses were also accepted with a significance level in the accepted range. Similar kinds of results were also evaluated by some previous researchers who only evaluated the direct impact of emotional exhaustion on the behavior of knowledge hiding (Zhao and Jiang, 2021), but the results of that research showed only the direct role of emotional exhaustion on knowledge hiding behavior, and the mediating role of other variables was studied in that research. Our research focused on factors of knowledge hiding, and it compartmentalized the cumulative impact of emotional exhaustion on these three factors of knowledge hiding, showing an equal impact on all.

The mediating effects of emotional exhaustion were also studied in this research. The mediating role of emotional exhaustion was suggested by Zhao and Jiang (2021). They found significant mediation of emotional exhaustion on knowledge hiding behaviors. Hypothesis indicated a strong mediation of emotional exhaustion between interpersonal injustice and knowledge hiding behaviors such as evasive hiding, playing dumb, and rationalized hiding. This kind of indirect relationship boosted the direct relationship of interpersonal injustice and knowledge hiding behaviors. Although the direct relationships were also significant, the mediator of emotional exhaustion facilitated the more toward knowledge hiding behaviors.

The last hypothesis was about the moderating role of high-performance work stress on interpersonal injustice and emotional exhaustion. Although the direct relationship of interpersonal injustice was significant toward emotional exhaustion, the moderator of high-performance work stress suggested that interpersonal injustice could lead to emotional exhaustion and the stress of performing in high-performance work systems would certainly worsen the scenario when injustice prevails and leads toward the exhaustion of workers. This also indicated that stress is the regulating factor while doing targeted task fulfillment. Previously, similar types of results were obtained in some studies (Siu, 2002; Golparvar et al., 2012; Liu et al., 2020), which indicated that high-performance work stress could further lead to other health-related exhaustions.

## Practical Implications

This study has offered few important practical implications. First, it is imperative for the organizations that they make efforts to ensure fairness in key operational activities. It will generate favorable outcomes such as knowledge sharing and enhanced productivity. Organizations can also promote interpersonal justice by undertaking various confidence-building exercises that are aimed at enhancing the interpersonal working relationships between the employees. Second, it is also important that organizations make sure that employees are not overburdened with the workload that they feel emotionally drained and exhausted as it might lead to negative outcomes such as knowledge hiding behaviors. Emotional exhaustion can be addressed by making sure that the employees are appreciated for their work, setting realistic work demands, ensuring fair and just practices, and promoting a culture that encourages work-life balance. Third, the organizations need to effectively address the work stressing high performance to avoid the knowledge hiding as a result of emotional exhaustion and organizational injustice. This can be guaranteed by developing comprehensive stress management and wellness programs that include the promotion of activities such as flexible and remote working, social bonding, workshops, and onsite employee counseling sessions.

## Limitations and Directions for Future Studies

Certain limitations were associated with this study. First, the methodological limitation lies with this study that quantitative analysis has been performed; however, many factors remain unexplored in a quantitative variable that can be investigated in the qualitative studies. Future studies should broaden the scope to mixed methods to yield more reliable results. Second, this study was undertaken in the Chinese cultural context. Further replication of this study in the context of other nations would certainly aid in enhancing the understanding of the various antecedents of knowledge hiding. Third, this study only observed the impact of interpersonal injustice on knowledge hiding and emotional exhaustion. Future studies can include other facets of organizational injustice such as procedural injustice, interactional injustice, and distributive injustice in order to broaden the understanding of the factors that influence knowledge hiding.

## Conclusion

In today’s competitive business environment, it becomes necessary for an organization to ensure that it retains the best talent. One way of achieving this is by making sure that the employees of the organization are treated with dignity and fairness so that they may develop a favorable perception about their workplace and thus, contribute toward the organization’s success by creating a culture of knowledge sharing. Therefore, this study was undertaken to assess and investigate the impact of interpersonal injustice on the three main facets of knowledge hiding, i.e., evasive hiding, playing dumb, and rationalized hiding. For this purpose, the employees working in the telecom sector of China were chosen as the respondents. The results indicated that interpersonal injustice had a positive relationship with evasive knowledge hiding behavior, playing dumb, and rationalized knowledge hiding behavior. Interpersonal injustice was also observed to have a positive impact on emotional exhaustion. Moreover, emotional exhaustion also had a positive relationship with all the aforementioned facets of knowledge hiding. Furthermore, emotional exhaustion mediated the relationship between interpersonal injustice and knowledge hiding (i.e., evasive hiding, playing dumb, and rationalized hiding), and high-performance work stress moderated the relationship between interpersonal injustice and emotional exhaustion.

## Data Availability Statement

The original contributions presented in the study are included in the article/supplementary material, further inquiries can be directed to the corresponding author/s.

## Ethics Statement

The studies involving human participants were reviewed and approved by the Hebei Finance University, China. The patients/participants provided their written informed consent to participate in this study. This study was conducted in accordance with the Declaration of Helsinki.

## Author Contributions

YC conceived, designed the concept, collected the data, wrote the manuscript, read, and agreed to the published version of the manuscript.

## Conflict of Interest

The author declares that the research was conducted in the absence of any commercial or financial relationships that could be construed as a potential conflict of interest.

## Publisher’s Note

All claims expressed in this article are solely those of the authors and do not necessarily represent those of their affiliated organizations, or those of the publisher, the editors and the reviewers. Any product that may be evaluated in this article, or claim that may be made by its manufacturer, is not guaranteed or endorsed by the publisher.
